# Virological Suppression and its Predictors Among HIV/AIDS Patients on Antiretroviral Therapy in Ethiopia: Systematic Review and Meta-analysis

**DOI:** 10.1093/ofid/ofae168

**Published:** 2024-03-21

**Authors:** Dagnachew Melak, Fekade Demeke Bayou, Husniya Yasin, Aregash Abebayehu Zerga, Birhanu Wagaye, Fanos Yeshanew Ayele, Natnael Kebede, Asnakew Molla Mekonen, Ahmed Hussien Asfaw, Shambel Ayichew Tsegaw, Mengistu Mera Mihiretu, Yawkal Tsega, Elsabeth Addisu, Niguss Cherie, Tesfaye Birhane, Zinet Abegaz, Abel Endawkie, Anissa Mohammed

**Affiliations:** Department of Epidemiology and Biostatistics, School of Public Health, Colleges of Medicine and Health Science, Wollo University, Dessie, Ethiopia; Department of Epidemiology and Biostatistics, School of Public Health, Colleges of Medicine and Health Science, Wollo University, Dessie, Ethiopia; Department of Epidemiology and Biostatistics, School of Public Health, Colleges of Medicine and Health Science, Wollo University, Dessie, Ethiopia; Department of Public Health Nutrition, School of Public Health, College of Medicine and Health Sciences, Wollo University, Dessie, Ethiopia; Department of Public Health Nutrition, School of Public Health, College of Medicine and Health Sciences, Wollo University, Dessie, Ethiopia; Department of Public Health Nutrition, School of Public Health, College of Medicine and Health Sciences, Wollo University, Dessie, Ethiopia; Department of Health Promotion, School of Public Health, College of Medicine and Health Sciences, Wollo University, Dessie, Ethiopia; Department of Health System Management, School of Public Health, College of Medicine and Health Sciences, Wollo University, Dessie, Ethiopia; Department of Public Health Nutrition, School of Public Health, College of Medicine and Health Sciences, Wollo University, Dessie, Ethiopia; Department of Nursing, Dessie Health Science College, Dessie, Ethiopia; Department of Health System Management, School of Public Health, College of Medicine and Health Sciences, Wollo University, Dessie, Ethiopia; Department of Health System Management, School of Public Health, College of Medicine and Health Sciences, Wollo University, Dessie, Ethiopia; Department of Reproductive and Family Health, School of Public Health, College of Medicine and Health Sciences, Wollo University, Dessie, Ethiopia; Department of Reproductive and Family Health, School of Public Health, College of Medicine and Health Sciences, Wollo University, Dessie, Ethiopia; Department of Reproductive and Family Health, School of Public Health, College of Medicine and Health Sciences, Wollo University, Dessie, Ethiopia; Department of Reproductive and Family Health, School of Public Health, College of Medicine and Health Sciences, Wollo University, Dessie, Ethiopia; Department of Epidemiology and Biostatistics, School of Public Health, Colleges of Medicine and Health Science, Wollo University, Dessie, Ethiopia; Department of Epidemiology and Biostatistics, School of Public Health, Colleges of Medicine and Health Science, Wollo University, Dessie, Ethiopia

**Keywords:** antiretroviral therapy, Ethiopia, HIV/AIDS, predictors, virological suppression

## Abstract

**Background:**

Achieving viral load suppression is crucial for the prevention of complications and deaths related to HIV infection. Ethiopia has embraced the worldwide 95-95-95 target, but there is no national representative information regarding virological suppression. Therefore, this review aims to determine the pooled virological suppression rate and identify the pooled effect of contributing factors of viral suppression for HIV-positive patients on antiretroviral therapy in Ethiopia.

**Methods:**

We systematically searched websites and databases, including online repositories, to obtain primary studies. Two reviewers assessed the quality of the included articles using the Newcastle-Ottawa Scale appraisal checklist. Publication bias was checked using Egger's regression test, the heterogeneity of the studies was assessed using *I*^2^ statistics and Q statistics, and a sensitivity analysis was performed to identify any outlier results in the included studies. The Der Simonian Laird random-effects model was used to estimate the overall proportion of viral suppression, and STATA 17 statistical software was used for all types of analysis.

**Results:**

A total of 21 eligible articles primarily conducted in Ethiopia using HIV program data were used for this quantitative synthesis. The overall pooled virological suppression rate was 71% (95% CI, 64%–77%). The pooled effects of poor adherence to ART (adjusted odds ratio [AOR], 0.33; 95% CI, 0.28–0.40), body mass index (18.5–24.9 kg/m^2^; AOR, 1.8; 95% CI, 1.37–2.36), disclosure (AOR, 1.41; 95% CI, 1.05–1.89), absence of opportunistic infection (AOR, 1.68; 95% CI, 1.43–1.97), and high baseline viral load count (AOR, 0.65; 95% CI, 0.52–0.81) were identified as significant predictors of viral suppression.

**Conclusions:**

The overall pooled percentage of virological suppression was low compared with the global target of viral suppression and the Ethiopian Public Health Institute report. Poor adherence, normal body mass index, disclosure, absence of opportunistic infection, and high baseline viral load count were factors contributing to viral suppression in Ethiopia. Responsible stakeholders should maximize their efforts to achieve the global target of virological suppression by addressing significant predictors.

The world has experienced a series of crises that have had a severe impact on individuals living with and affected by HIV in the past 2 years, particularly during the coronavirus disease 2019 (COVID-19) era [1]. These crises have not only hindered the global response to the AIDS pandemic but also exacerbated existing socioeconomic inequalities within and between countries, particularly in Sub-Saharan Africa [1–3]. The COVID-19 pandemic has caused significant disruptions to essential HIV treatment and prevention services, derailing the progress made in HIV response [1, 4].

It is our collective responsibility to put an end to the AIDS epidemic, honoring the memory of the 39 million individuals who have lost their lives to this disease [2, 5]. The Joint United Nations Program on HIV/AIDS (UNAIDS) set a global target in December 2020, the 95-95-95 goal, aiming to achieve the following by 2025: 95% of all people with HIV will be aware of their HIV status, 95% will receive consistent antiretroviral therapy, and 95% of those on treatment will achieve viral suppression [6]. However, the current reality is that only three-quarters of people with HIV have access to antiretroviral treatment, and 52% of children with HIV have access to life-saving medication [1]. Shockingly, ∼10 million adult individuals still lack access to treatment, and the gap in HIV treatment coverage between children and adults is widening instead of narrowing [1, 7].

Ethiopia has embraced the UNAIDS target as a crucial component of its efforts to eliminate HIV/AIDS epidemics by 2030. The country has been collaborating with numerous partners and stakeholders to enhance disease detection, viral load testing, and adherence to antiretroviral therapy [8–10]. Consequently, the national prevalence of HIV infection among individuals aged 15–49 has decreased to 0.9%, although variations persist based on gender, location, and specific population groups, with higher rates among women and in urban areas [11].

Achieving viral load suppression (VLS) is crucial for the effective treatment and prevention of complications and deaths related to HIV infection [7, 12]. The World Health Organization (WHO) recommends regular viral load testing as the preferred method for monitoring and confirming treatment failure [13]. Virological suppression is defined as when the viral load count drops below 1000 copies/mL of blood or becomes undetectable after a sufficient duration of antiretroviral therapy (ART), possibly within 6 months of initiation [13].

A longitudinal analysis conducted across multiple countries in Sub-Saharan Africa revealed that the rates of virological suppression range from 90% to 93% [14]. In Rwanda, the rate is 83% [15], while in Cameroon it is 79.4% [16]; in Nigeria, it is 80.2% [17], and in Tanzania, it is 94% [18]. In Ethiopia, the virological suppression rates vary, with Hawassa reporting 40.9% [19], West Gojjam reporting 51.73% [20], North Shewa zone reporting 72% [21], and Debre Markos reporting 92% [22]. These variations in the reports highlight the need for nationally representative data on virological suppression in Ethiopia. It is critical to continue concerted efforts to address the uneven distribution of HIV infection and work toward achieving global targets uniformly within the remaining time frame [10].

In order to facilitate the establishment of a bold future goal, it is essential to analyze the pooled prevalence of virological suppression and the factors that contribute to it within the Ethiopian context. Therefore, the objectives of this review are to determine the overall national rate of virological suppression and analyze the contextual predictors of virological suppression using internationally recognized frameworks of key performance indicators. The findings of this study will provide valuable insights for health care providers and assist the country in sustaining the achievements of virological suppression while addressing areas of improvement to achieve the goal of ending AIDS by 2030.

## METHODS

### Reporting

We strictly followed the Preferred Reporting Items for Systematic Reviews and Meta-analyses (PRISMA) guidelines [23] with a supplementary file research checklist (Supplementary Table 1). The review began on May 14, 2023, and was completed on July 11, 2023, including the date for formulation of the research question and the preliminary search. We wrote a detailed protocol of the systematic review and meta-analysis before the review started and registered it with the Prospero database (registration number CRD42023434248; https://www.crd.york.ac.uk/prospero/#recordDetails).

### Inclusion and Exclusion Criteria

The articles selected for this systematic review and meta-analysis consisted of cohort, case‒control, and cross-sectional studies. Those studies reported the proportion of viral suppression at various time intervals and included adjusted effect measures of factors associated with virological suppression. Additionally, the studies had to be conducted in Ethiopia using national HIV program data. Studies that lacked full-text access and requiring subscription, qualitative studies, and conference proceedings without full-text reports were excluded from the analysis.

### Search Strategy

Website searches (Google, Google Scholar, and registries) and database searches (PubMed, Science Direct, and Hinari [research4life]) were conducted to find research articles. The search strategy used in PubMed was [((virological) OR (viral)) AND ((suppression) OR (re-suppression)) AND ((predictors) OR (factors) OR (determinants)) AND ((HIV) OR (AIDS)) AND ((“antiretroviral therapy”) OR (ART) OR (HAART)) AND (Ethiopia)]; similar search terms were used for Hinari. In addition, Ethiopian university online repositories (University of Gondar, Addis Ababa University, Hawassa University, and Bahirdar University) were searched. Endnote 20 reference manager software was used to manage duplicated references and for citations in the manuscript. All accessed databases were searched from June 18 to 23, 2023.

### Outcome Measurement/Ascertainment

Virological suppression was considered when viral load copies become <1000 copies/mL of blood after initiation of ART [13].

### Data Extraction Process

Data from each study were independently extracted by 2 reviewers (A.M. and H.Y.) and cross-checked by both reviewers. Any discrepancies were resolved through revision. The extracted data included the first author and year of publication, sample size, number of individuals with the outcome of interest, study design, study population with sample size, geographical location, funding information, and response rate. The data were extracted from June 25 to July 2, 2023.

### Quality Assessment

Two authors (A.M., Y.T.) independently evaluated the quality of the articles using the Newcastle‒Ottawa Scale, which is a tool for assessing the quality of cross-sectional, case‒control, and cohort studies [24].

The criteria for cross-sectional studies consisted of 3 sections. The first section focused on selection (rated on a scale of up to 5 stars), which considers the representativeness of the average general population, justifiable sample size, satisfactory response rate, and the use of a validated measurement tool for ascertainment of the exposure (risk factors). The second section assessed the comparability of the study (rated on a scale of up to 2 stars), which considers whether subjects in different outcome groups are comparable based on the study design or analysis and whether confounding factors are controlled for. The third section assessed the outcome (rated on a scale of up to 3 stars), which includes assessment of outcome (independent blind assessment, record linkage, self-report, and no description) and the statistical test used to describe the data (whether it is clearly described and appropriate and the measurement of the association is presented, including confidence intervals and probability level [*P* value]).

For case–control studies, the criteria included selection, which was evaluated with a maximum of 4 stars by considering the adequacy of the case definition and representativeness of the cases; selection of controls and definition of controls’ comparability, assessed with a maximum of 2 stars by comparability of cases and controls on the basis of the design or analysis; and exposure, graded with a maximum of 4 stars by considering the method of ascertainment of exposure and use of the same method of ascertainment for cases and controls.

Cohort studies had criteria that included selection, graded with up to 6 stars by considering the representativeness of the exposed cohort, selection of the nonexposed cohort, ascertainment of exposure, and demonstration that the outcome of interest was not present at the start of the study; comparability, graded with up to 2 stars by considering the comparability of the cohorts on the basis of the design or analysis; outcome, graded with up to 5 stars by considering the methods of assessment of outcome, whether follow-up was long enough for outcomes to occur, and adequacy of follow-up of cohorts.

Cross-sectional studies that scored ≥6, case–control studies that scored ≥7, and cohort studies scored that ≥9 on the quality assessment criteria were included in the review (Supplementary Table 2). In cases where there was disagreement between the 2 assessors, the process was repeated and resolved with the involvement of a third reviewer (M.M.).

### Statistical Analysis and Data Synthesis

Publication bias was assessed using funnel plots, or Egger's test for a more objective evaluation. The heterogeneity of the studies was assessed using *I*^2^ and Q statistics, and a sensitivity analysis was performed to identify any outlier results in the included studies. The Der Simonian Laird random-effects model was used to estimate the overall proportion of viral suppression. A subgroup analysis was conducted based on the geographical location of the study, type of antiretroviral therapy regimen, sample size, study population by age, and study design to examine variations in outcomes. STATA 17 statistical software was used for analysis. A cumulative meta-analysis was conducted to indicate temporal variation of the virological suppression rate by including studies sequentially from their year of publication.

## RESULTS

**Figure 1.**
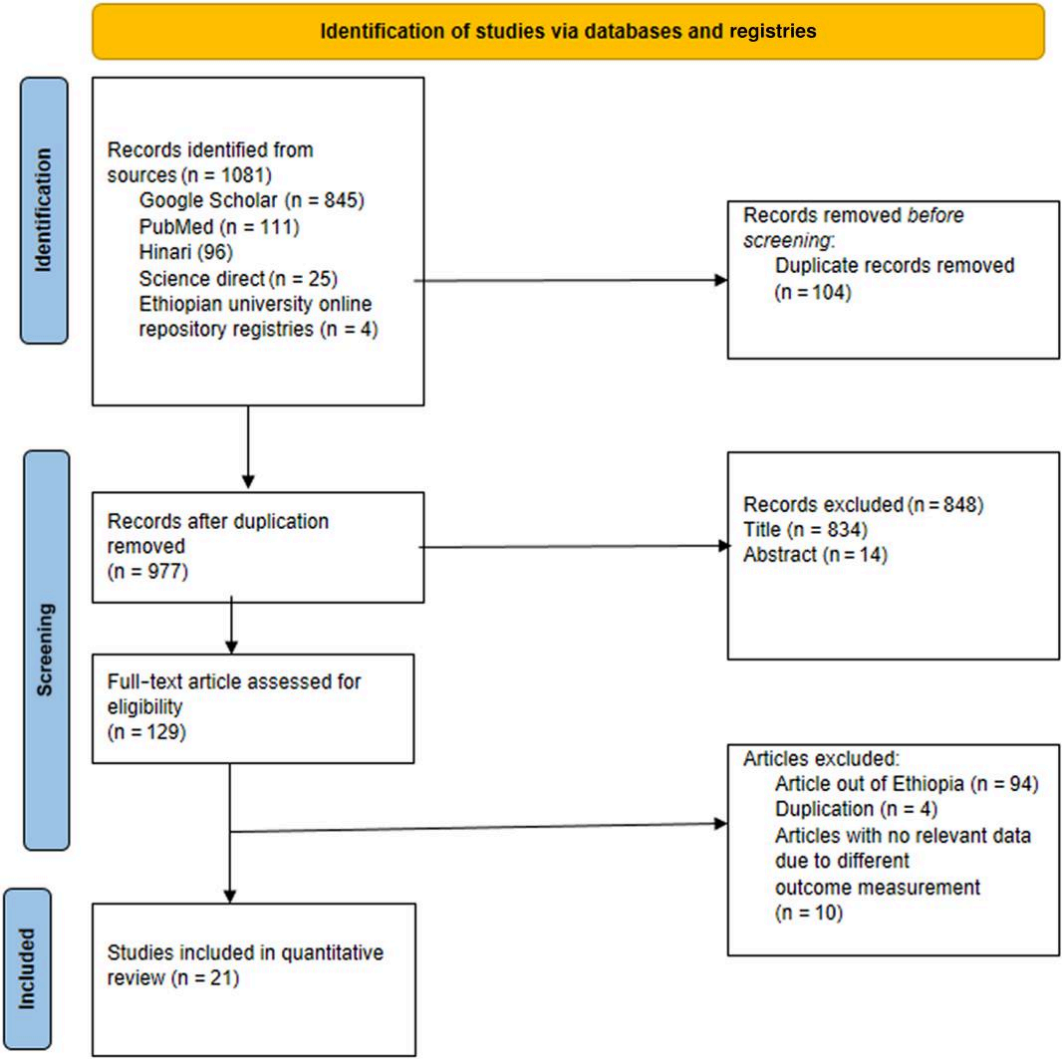
PRISMA flow-chart diagram describing the screening and selection of studies for systematic review and meta-analysis in Ethiopia.

A comprehensive search of databases and websites, including Google Scholar (n = 846), PubMed (n = 111), science direct (n = 25), Hinari research4life (n = 96), Ethiopian university online repositories (Hawassa University n = 1, Bahirdar University n = 1, and Addis Ababa University n = 1), and other sources (n = 1), yielded a total of 1081 articles. After removing 104 duplicate articles, 977 articles remained. Among these, 848 articles were excluded based on a lack of matching titles and abstracts. Additionally, 108 articles were excluded after a full-text review due to differences in outcome variable measurement (n = 10), duplication (n = 4), and being conducted outside Ethiopia (n = 94). Finally, a total of 21 articles were included for quantitative review (Figure 1).

### Characteristics of the Included Articles in Ethiopia

The studies found through database searching and included for the current review were performed between 2016 and 2022 and published between 2018 and 2023. Regarding the study area/region, 7 articles were conducted in the Amhara region [20, 25–30], 6 studies in the Oromia region [21, 31–35], 5 articles in the South Nation and Nationalities and Peoples of Ethiopia (SNNPE) region [19, 36–39], 1 study in the Tigray region [40], and 2 studies in the Addis Abeba city administration [41, 42].

Ten articles were conducted using a cross-sectional study design [19, 25, 28, 32–35, 37, 39, 40], 2 studies were conducted using a case–control study design [31, 41], 8 studies were conducted using a retrospective cohort study [20, 21, 26, 27, 29, 30, 36, 42], and 1 study was conducted using a prospective cohort study [38]. Nine studies were conducted on first-line ART patients [21, 26, 31, 33, 34, 36, 37, 38, 42], 9 studies were conducted on both first- and second-line ART patients [19, 20, 25, 28, 29, 32, 35, 39, 40, 41], and 2 were conducted on second-line ART patients [27, 30]. The minimum sample size was 152 in the study in Arba Minch [38], and the maximum sample size was 19 525 in the study in the Tigray region (Table 1) [40].

**Table 1.** Characteristics of the Included Studies in the Systematic Review and Meta-analysis in Ethiopia, 2023

Author Name/Publication Year	Study Period	Study Region/Area	Study Setting	Population	ART Regimen	Sample Size	Response Rate, %	Study Design	Funding Source
Ali et al./2019 [21]	2011 −2013	Oromia	Health center	Adult	First-line ART	243	100	Retrospective cohort	Saint Paul's Hospital, Ethiopia
Anito et al./2022 [19]	May to June 2021	SNNPE	Hospital	All ART patients	All ART regimens	342	100	Cross-sectional	Not reported
Atnafu et al./2022 [20]	2016–2020	Amhara	All health facilities	Adult	All ART regimens	347	100	Retrospective cohort	Not reported
Berihun et al./2023 [25]	March to June 2022	Amhara	APHI	Children	All ART regimens	522	100	Cross-sectional	Not reported
Diress et al./2020 [26]	March to May 2019	Amhara	Hospital	All ART patients	First-line ART	235	100	Retrospective cohort	Woldia University, Ethiopia
Erjino et al./2023 [36]	2016–2021	SNNPE	Hospital	Adult	First-line ART	297	100	Retrospective cohort	Not reported
Fenta/2021 [37]	July to Dec 2019	SNNPE	Hospital	Children	First-line ART	273	100	Cross-sectional	Hawassa University, Ethiopia
Kolako/2019 [39]	March 2019	SNNPE	Hospital	Adult	All ART regimens	367	98	Cross-sectional	Not reported
Melku et al./2020 [28]	Feb to April 2018	Amhara	Hospital	Adult	All ART regimens	323	100	Cross-sectional	Not reported
Minyichil/2022 [29]	2017–2021	Amhara	All health facilities	All ART patients	All ART regimens	546	96	Retrospective cohort	Not reported
Sado et al./2022 [32]	June to November 2019	Oromia	Hospital	Adult	All ART regimens	430	100	Cross-sectional	Not reported
Sorsa/2018 [33]	May–August 2017	Oromia	Hospital	Children	First-line ART	183	100	Cross-sectional	Self
Waju et al./2021 [34]	March 2019	Oromia	All health facilities	Adult	First-line ART	669	100	Cross-sectional	Not reported
Wedajo et al./2021 [30]	2016–2019	Amhara	Hospital	Adult	Second-line ART	642	100	Retrospective cohort	Bahir Dar University, Ethiopia
Melak et al./2023 [30]	2018–2022	Amhara	Hospital	Adult	Second-line ART	364	100	Retrospective cohort	Wollo University, Ethiopia
Desta et al./2020 [40]	2015–2019	Tigray	THRI	All ART patients	All ART regimens	19 525	100	Cross-sectional	Not reported
Hussen et al./2019 [38]	2017–2018	SNNPE	Hospital	Adult	First-line ART	152	100	Prospective cohort	Arba Minch University. Ethiopia
Dires et al./2021 [41]	2021	Addis Abeba	Hospital	Youth	All ART regimens	192	100	Case–control	Addis Ababa University, Ethiopia
Sosna/2021 [42]	2018–2019	Addis Abeba	All health facilities	Adult	First-line ART	356	93	Retrospective cohort	Not reported
Haile et al./2021 [35]	August–October 2020	Oromia	All health facilities	Adult	All ART regimens	424	100	Cross-sectional	Not reported
Jaleta et al./2022 [31]	2015–2020	Oromia	All health facilities	Adult	First-line ART	347	100	Case–control	Oromia Regional Health Bureau (ORHB)

Abbreviations: ART, antiretroviral therapy; SNNPE, South Nation and Nationalities and Peoples of Ethiopia; THRI, Tigray Health Research Institute.

### Publication Bias

**Figure 2.**
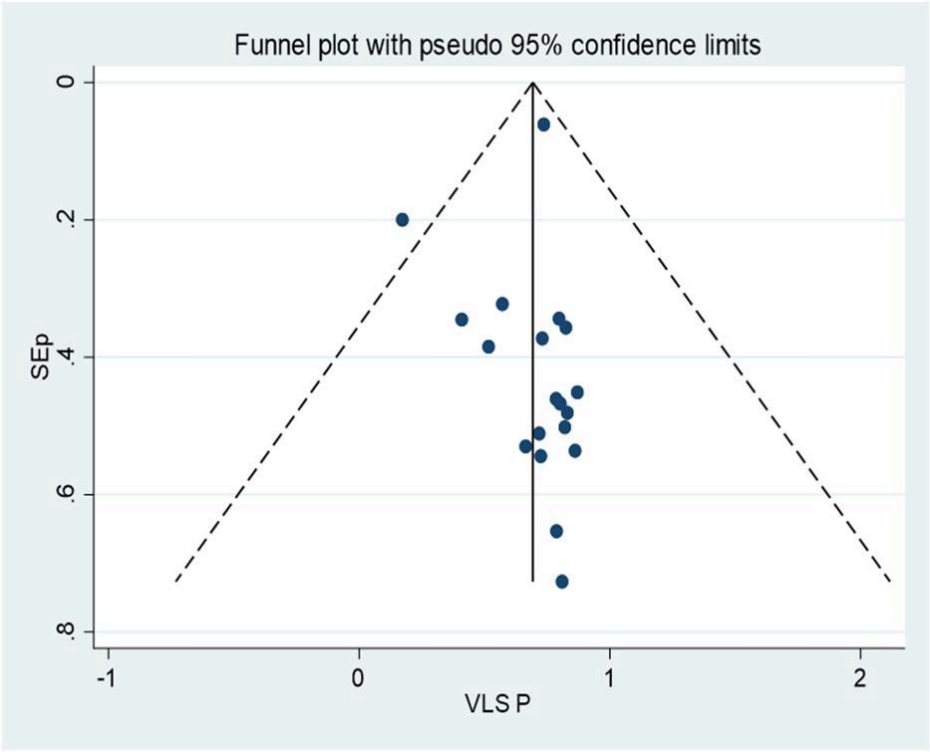
Funnel plot, in which the vertical line indicates the effect size, whereas the diagonal line indicates the precision of individual studies with a 95% confidence limit. VLS P, viral load suppression proportion.

Figure 2 shows a funnel plot for HIV viral load suppression, which indicates symmetries. Egger's test showed no small-study effects, with a *P* value of .747.

### Meta-analysis of the Proportion With Virological Suppression in Ethiopia

**Figure 3.**
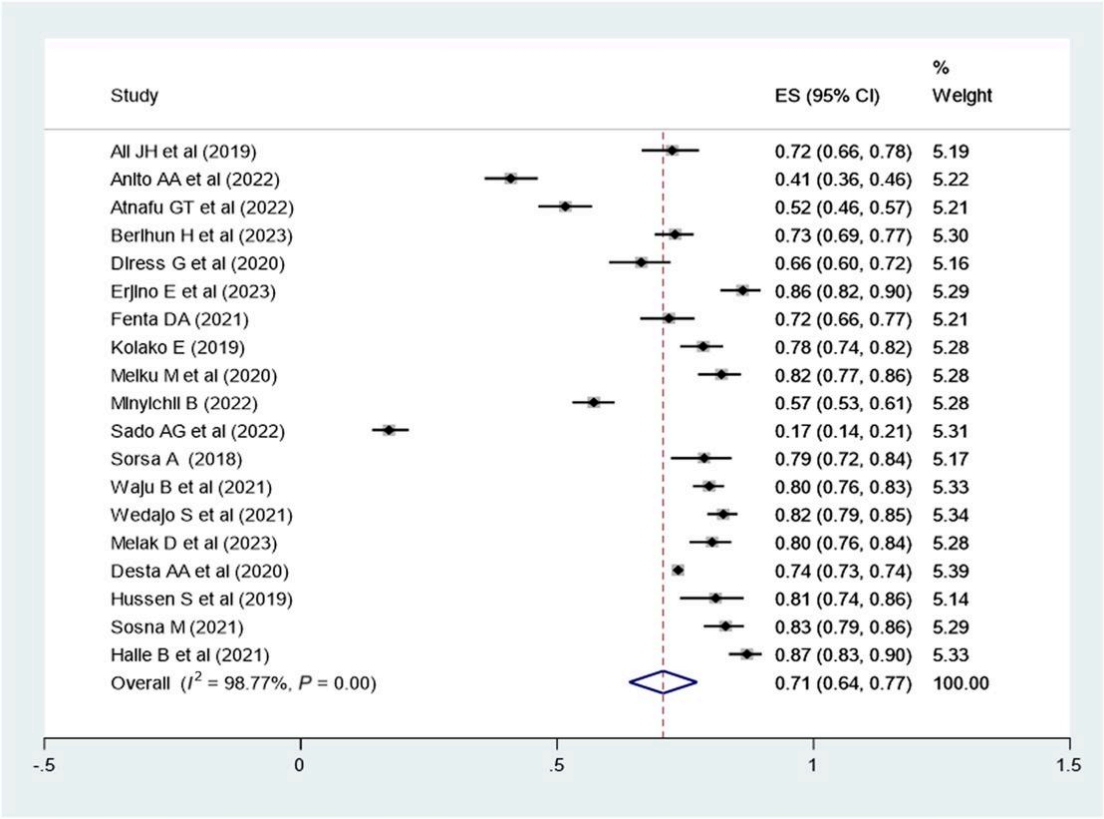
Forest plot of the proportion of virological suppression in Ethiopia and its 95% CI. The midpoint of each line illustrates the estimated proportion of VL in each study. The diamond shows the pooled proportion.

Virological suppression rate (suppressed: <1000 copies/mL of blood; or unsuppressed: ≥1000 copies/mL of blood) was determined according to the Ethiopian context. To determine the pooled proportion of viral suppression, 19 studies were included, with a total sample size of 26 240 participants. By definition, the pooled prevalence of virological suppression in the Ethiopian context was 71% (95% CI, 64%–77%; *I*^2^ = 98.77; Q statistics *P* ≤ .01) (Figure 3).

### Subgroup Analysis of Virological Suppression in Ethiopia

**Figure 4.**
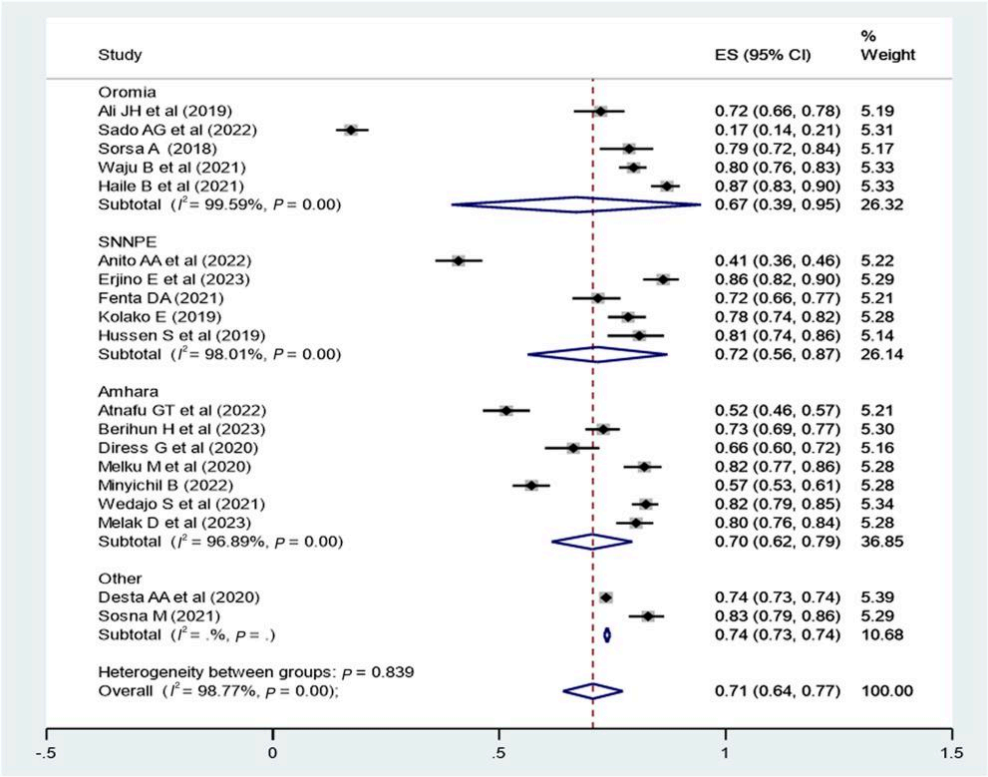
Subgroup analysis by region of Ethiopia to determine the pooled proportion of virological suppression in each region. The midpoint of each line illustrates the estimated proportion of VL in each study. The diamond shows the pooled proportion.

A subgroup analysis was employed by region (Figure 4), study population, study design, and ART regimen. The virological suppression rate was 62% in the Oromia region, 72% in the SNNPE region, 70% in the Amhara region, and 74% in other regions (Tigray and Adis Ababa). The pooled virological suppression rate among adults was found to 73% (95% CI, 61%–85%), whereas in children it was 74% (95% CI, 70%–78%). The pooled virological suppression rate for patients on first-line ART was 78% (95% CI, 73%–82%), whereas for those on second-line ART it was 82% (95% CI, 79%–84%) (Table 2).

**Table 2.** Subgroup Analysis of the HIV Virological Suppression Percentage by Region, Study Population, Study Design, and ART Regimen Type in Ethiopia, 2023

Subgroup Analysis	No. of Studies	Pooled Percentage of VLS	95% CI of *P*	Heterogeneity *I*^2^, % (*P* Value)
By region				
Oromia	5	67	39–95	99.6
SNNPE	5	72	56–87	98.01
Amhara	7	70	62–79	96.89
Other^a^	2	74	73–74	0.0
By study population				
Adults	12	73	61–85	99.11
All ART patients	4	60	45–75	98.56
Children	3	74	70–78	0.0
By study design				
Cohort study	9	73	65–81	96.60
Cross-sectional study	10	68	57–79	99.25
By ART regimen				
First-line	8	78	73–82	84.86
First- and second-line	8	62	50–75	99.38
Second-line	2	82	79–84	0.0
Overall pooled percentage	19	71	64–77	98.8 (.839)

Abbreviations: ART, antiretroviral therapy; SNNPE, South Nation and Nationalities and Peoples of Ethiopia; VLS, viral load suppression.

^a^Addis Ababa and Tigray region.

### Cumulative Proportion of Virological Suppression Rate Over the Publication Years (2018–2023) in Ethiopia

**Figure 5.**
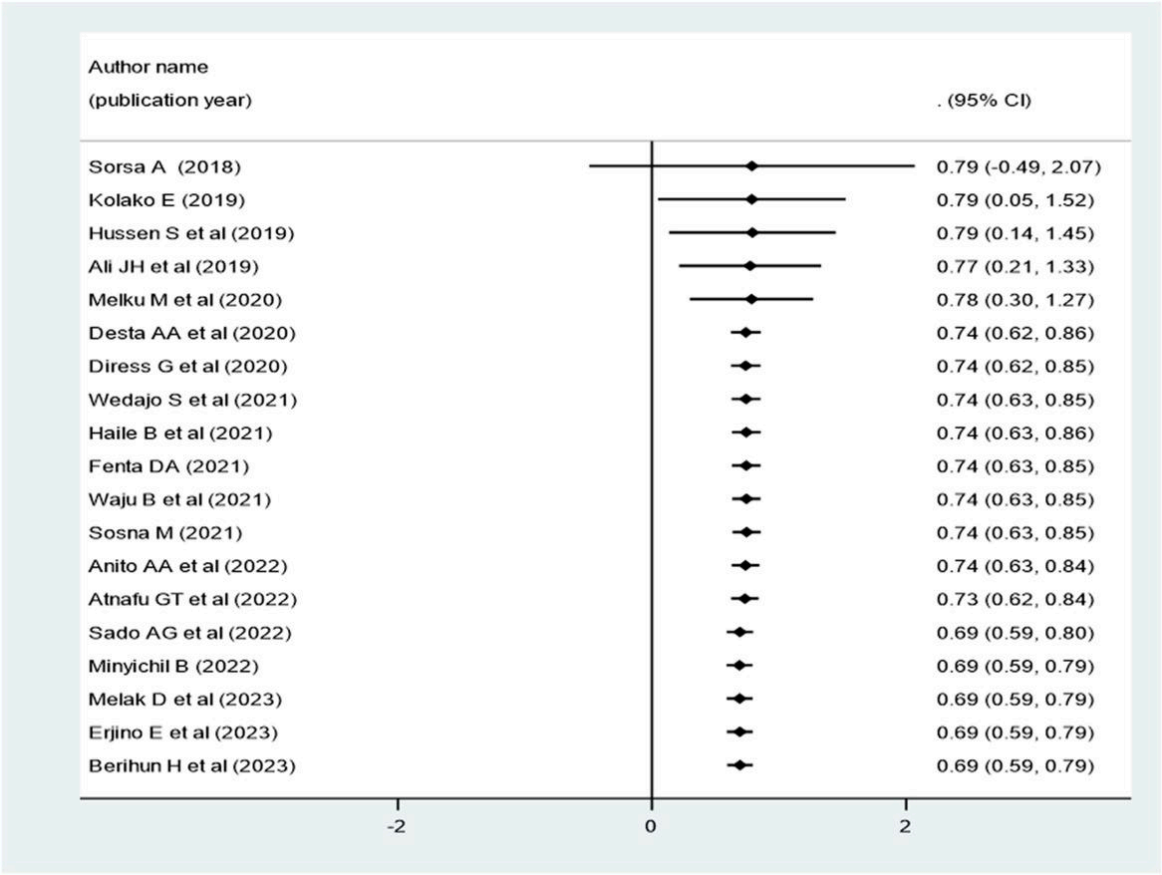
Forest plot for the cumulative proportion of virological suppression over the publication year of individual articles conducted in Ethiopia (2018–2023).

The cumulative proportion of virological suppression in Ethiopia was higher from 2018 to 2020 but constantly decreased from 2020 to 2023 (Figure 5).

### Systematic Review of Factors Associated With Virological Suppression in Ethiopia

Different factors, such as sociodemographic, clinical, and treatment-related factors, contribute to virological suppression.

#### Sociodemographic Factors Associated With Virological Suppression

Based on the primary study report, being female was positively associated with virological suppression (adjusted hazard ratio [AHR], 1.50; 95% CI, 1.05–2.15 [20]; adjusted relative risk [ARR], 1.18; 95% CI, 1.017–1.192 [26]; adjusted odds ratio [AOR], 1.9; 95% CI, 1.04–3.48 [37]). Another study revealed a similar finding, in which being male had a positive association with viral suppression status (AOR, 1.27; 95% CI, 1.18–1.37) [40]. Different age categories also showed an association with viral suppression: ages 15–19 years (AOR, 4.86; 95% CI, 3.86–6.12), 20–24 years (AOR, 1.96; 95% CI, 1.57–2.45), 25–29 years (AOR, 1.79; 95% CI, 1.55–2.08), 30–34 years (AOR, 1.46; 95% CI, 1.29–1.65), 35–39 years (AOR, 1.43; 95% CI, 1.27–1.61), 40–44 years (AOR, 1.22; 95% CI, 1.08–1.39), 45–49 years (AOR, 1.22; 95% CI, 1.06–1.40) were positively associated with viral suppression status compared with older age groups 50+ years [40]. In the primary study, having formal education in primary school (ARR, 1.38; 95% CI, 1.032–1.841) and secondary and above (ARR, 1.65; 95% CI, 1.253–2.164) were positively associated with viral suppression [26].

#### Clinical Factors Associated With Virological Suppression

In the primary study, malnourished patients were less likely to achieve viral suppression (AOR, 0.565; 95% CI, 0.329–0.971) [19], and patients with a BMI between 18.5 and 24.9 kg/m^2^ were more likely to have virological resuppression (AHR, 1.42; 95% CI, 1.03–1.95) [27]. Poor adherence (AOR, 0.504; 95% CI, 0.287–0.886) [19] and another study [40] showed that poor adherence was more likely to lead to suppression (AOR, 2.56; 95% CI, 1.97–3.33). CD4 count <200 cells/mm^3^ (AOR, 0.149; 95% CI, 0.071–0.314) [19] and CD4 count >350 cells/mm^3^ (AHR, 1.98; 95% CI, 1.12–3.51) [20] showed a positive association with viral suppression. The presence of opportunistic infection (AHR, 1.85; 95% CI, 1.06–3.24) showed a positive association with virological suppression status [20].

#### Treatment-Related Predictors of Virological Suppression

NVP-based (AOR, 0.125; 95% CI, 0.034–0.464) and EFV-based (AOR, 0.223; 95% CI, 0.063–0.0795) ART regimens were less likely to suppress viral load than PI-based regimens [19]. Another study showed that patients on second-line regimens were more likely to have virological suppression than those on first-line regimens (AOR, 8.98; 95% CI, 2.64–30.58) [35]. Patients who used cotrimexazole preventive therapy (CPT; AHR, 1.997; 95% CI, 1.108–3.600 [38]; AOR, 2.6; 95% CI, 1.23–5.48 [31]) and isoniazid preventive therapy (IPT; AHR, 3.09; 95% CI, 1.72–5.53 [38]) were more likely to achieve virological suppression compared with nonusers.

### Meta-analysis of Factors Associated With Virological Suppression in Ethiopia

**Figure 6.**
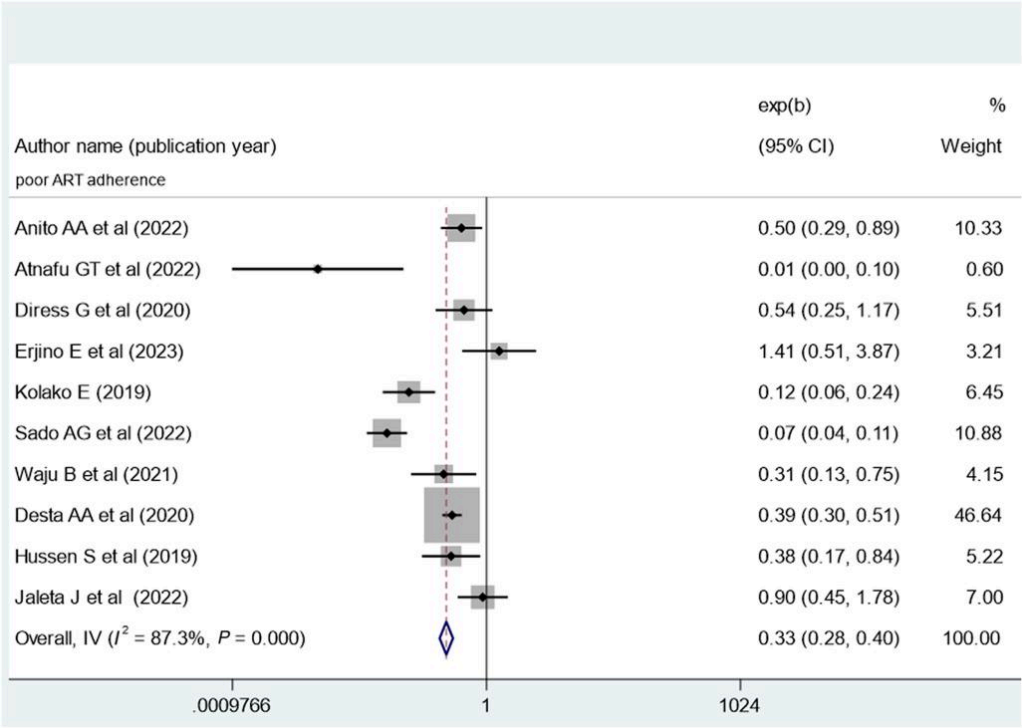
Forest plot of the adjusted odds ratios with corresponding 95% CIs of studies on the association of poor ART adherence and virological suppression in Ethiopia. ART, antiretroviral therapy.

**Figure 7.**
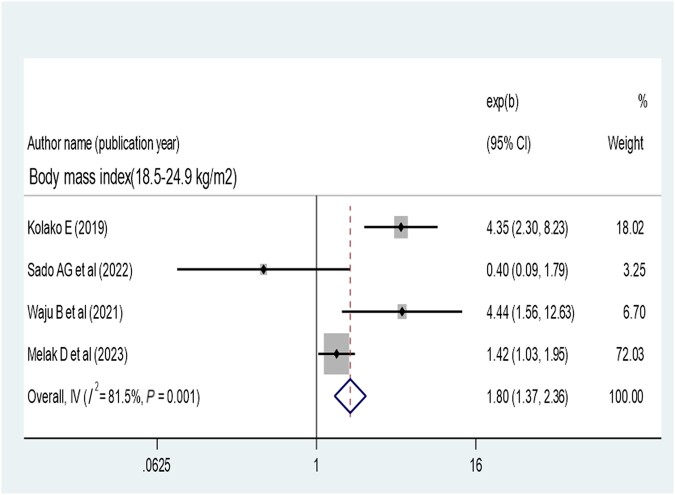
Forest plot of the adjusted odds ratios with corresponding 95% CIs of studies on the association of body mass index and virological suppression.

**Figure 8.**
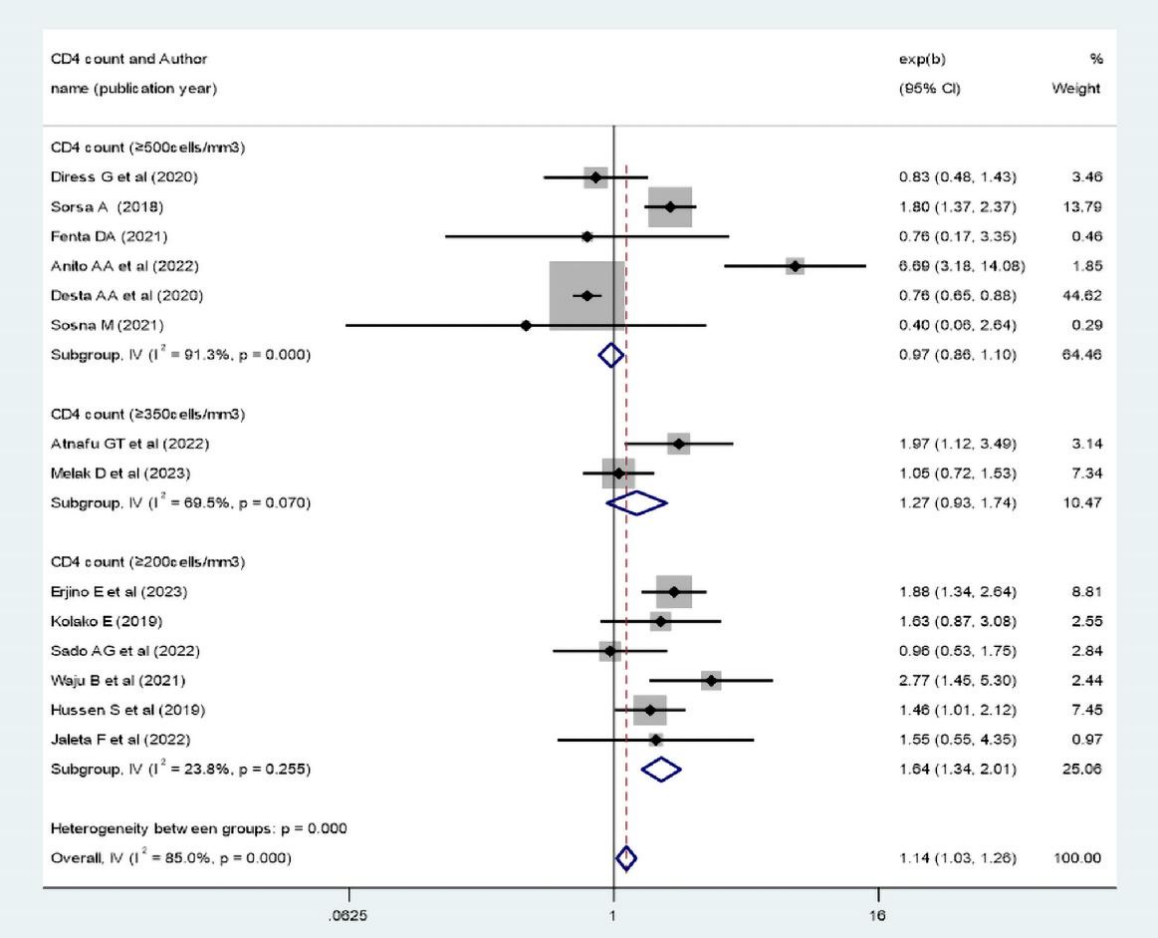
Forest plot of the adjusted odds ratios with corresponding 95% CIs of studies on the association of CD4 count and virological suppression. CD4, cluster of differentiation.

The pooled effect of patient adherence to ART showed that patients with poor adherence were 0.33 times less likely to achieve virological suppression (AOR, 0.33; 95% CI, 0.28–0.40) (Figure 6). The pooled effect of BMI (18.5–24.9 kg/m^2^) showed a positive association with virological suppression (AOR, 1.8; 95% CI, 1.37–2.36) (Figure 7). The pooled effect of CD4 count ≥200 cells/mm^3^ indicated a 1.64 times higher rate of virological suppression compared with CD4 <200 cells/mm^3^ (AOR, 1.64; 95% CI, 1.34–2.01) (Figure 8).

**Figure 9.**
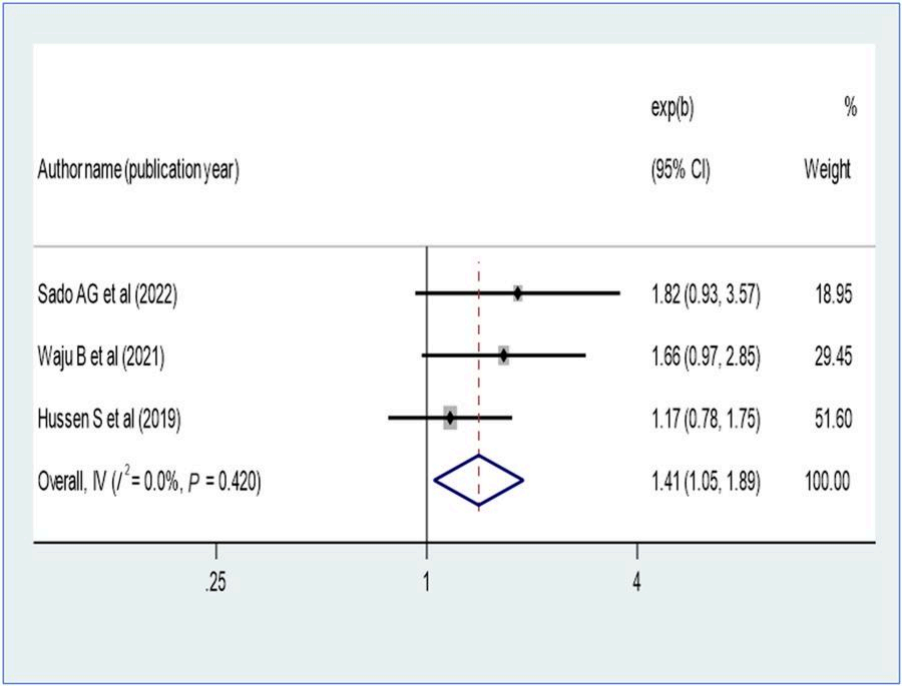
Forest plot of the adjusted odds ratios with corresponding 95% CIs of studies on the association of disclosure of HIV status and virological suppression.

**Figure 10.**
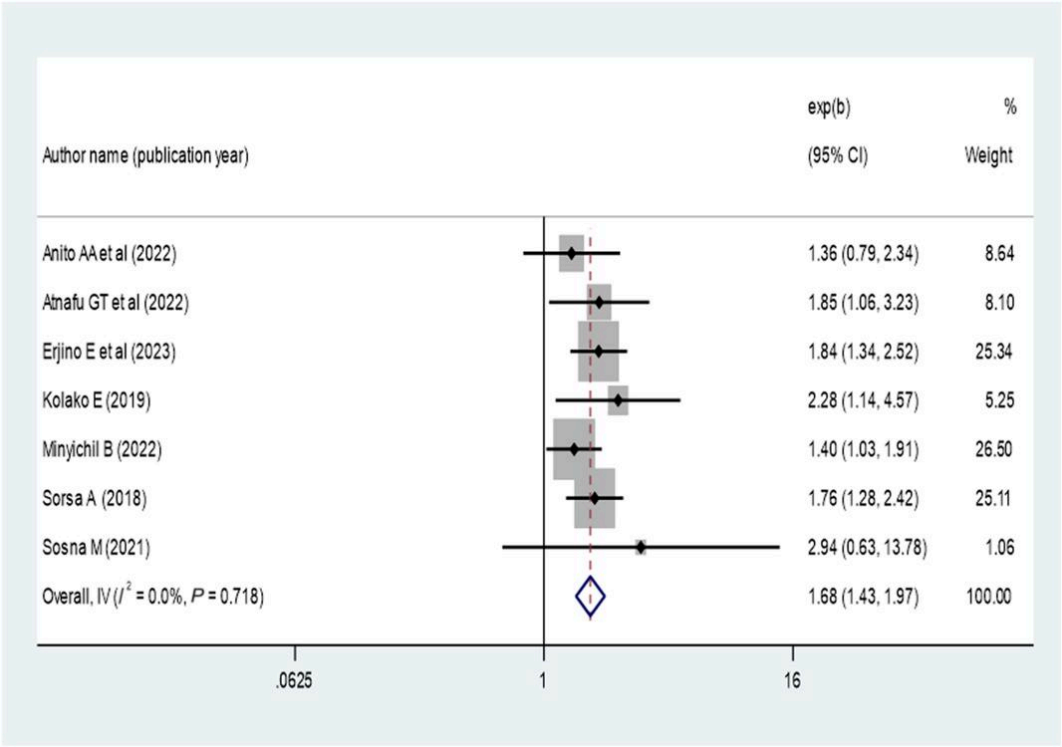
Forest plot of the adjusted odds ratios with corresponding 95% CIs of studies on the association of absence opportunistic infection and virological suppression.

**Figure 11.**
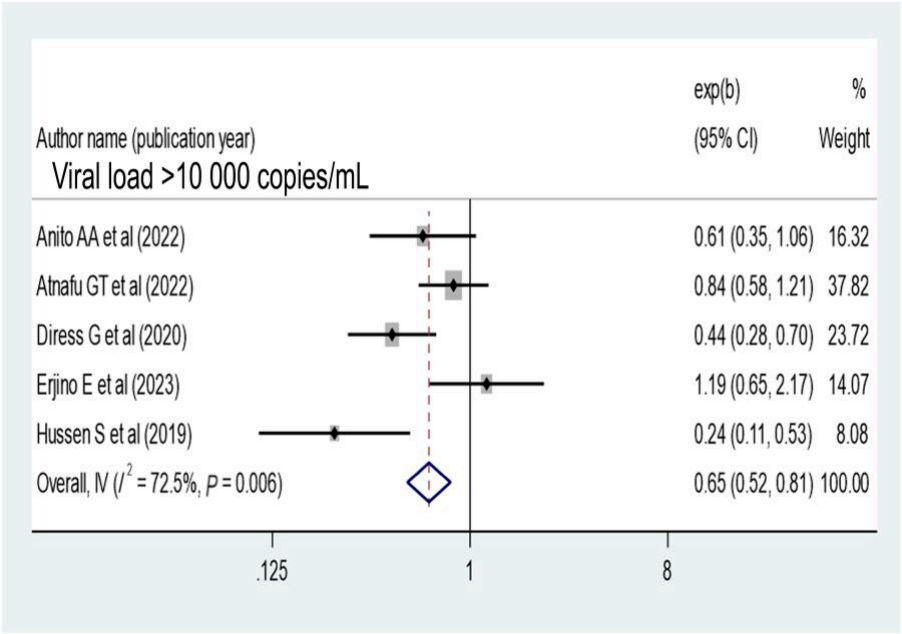
Forest plot of the adjusted odds ratios with corresponding 95% CIs of studies on the association of baseline viral load ≥10 000 copies/mL and virological suppression.

Patients who disclosed their HIV status were 1.41 times more likely to have virological suppression (AOR, 1.41; 95% CI, 1.05–1.89) (Figure 9). Opportunistic infection also determines virological suppression; the absence of opportunistic infection increases virologic suppression by 1.68-fold as compared with patients with opportunistic infection (AOR, 1.68; 95% CI, 1.43–1.97) (Figure 10). Baseline viral load count showed a significant association with viral suppression; viral load count ≥10 000 copies/mL of blood had 35% lower virologic suppression as compared with its counterparts (AOR, 0.65; 95% CI, 0.52–0.81) (Figure 11). In this study, the pooled effect of WHO stage did not show a significant association with virological suppression. A summary of the findings can be found in Table 3.

**Table 3.** Summary of Pooled Effect of Factors Associated With Virological Suppression in Ethiopia, 2023

Variables	No. of Studies	AOR	95% CI of AOR	Heterogeneity *I*^2^, % (*P* Value)
Poor adherence	10	0.33	0.28–0.40	87.3 (.000)
BMI (18.5–24.9 kg/m^2^)	4	1.80	1.37–2.36	81.5 (.001)
CD4 count ≥200 cells/mm^3^ at baseline	6	1.64	1.34–2.01	23.8 (.255)
CD4 count ≥350 cells/mm^3^ at baseline	2	1.27	0.93–1.74	69.5 (.07)
CD4 count ≥500 cells/mm^3^ at baseline	6	0.971	0.86–1.10	91.3 (.000)
Having disclosed HIV status	3	1.41	1.05–1.89	0.00 (.42)
Absence of opportunistic infection	6	1.68	1.43–1.97	0.00 (.718)
Viral load ≥10 000 copies at baseline	5	0.65	0.52–0.81	72.5 (.006)
WHO stage III/IV	7	0.98	0.86–1.13	70.9 (.002)

Abbreviations: AOR, adjusted odds ratio; ART, antiretroviral therapy; BMI, body mass index; WHO, World Health Organization.

## DISCUSSION

The objectives of this systematic review and meta-analysis were to determine the pooled proportion of virological suppression and investigate the pooled impact of factors contributing to virological suppression using primary studies conducted using HIV program data in Ethiopia. Based on our analysis of data from 19 primary studies conducted in Ethiopia, the pooled prevalence of virological suppression was 71% (95% CI, 64%–77%). This estimate is lower compared with the global target for virological suppression in controlled patients [5], multicounty longitudinal cohort analysis studies and meta-analyses of virological success rates in Sub-Saharan Africa [14, 43], a randomized controlled trial in Botswana [44], and a recent EPHI report in Ethiopia.

The variation in measurement of viral suppression in different contexts may explain this discrepancy. The low rate of virological suppression in this study could be attributed to an increased rate of treatment failure, as indicated by a recent meta-analysis in Ethiopia [45], as well as the impact of the COVID-19 pandemic on HIV care and treatment [49]. However, the findings of this study align with a nationwide estimate of virological suppression in Cameroon [46]. It is worth noting that the finding of this review is higher than the results reported in some studies conducted in certain regions of Ethiopia [19, 20].

The cumulative meta-analysis conducted in this study indicates a decline in the cumulative rate of virological suppression from 2020 to 2023. This decrease could be attributed to various challenges faced in Ethiopia. One of the significant challenges is the COVID-19 pandemic, which has had a profound impact on all aspects of HIV service delivery [49]. This includes difficulties in scheduling appointments for medication refills, difficulties with clinical and laboratory follow-ups for routine viral load testing, and an increase in the number of individuals lost to ART follow-up [47, 48]. Additionally, health care facilities have prioritized the response to COVID-19, leading to a disruption in routine HIV services [49], and the COVID-19 pandemic has had a detrimental effect on HIV treatment and prevention services globally [1, 4].

This review suggests that Ethiopia has not yet reached the global target of achieving virological suppression in 95% of patients on ART. In order to enhance the likelihood of achieving positive virological outcomes, it is necessary to address various factors and challenges. This systematic review and meta-analysis found that factors such as patient adherence to ART, body mass index, baseline CD4 count, disclosure, opportunistic infections, and baseline viral load count had a significant pooled impact on virological suppression in Ethiopia. By focusing on these factors, improvements can be made to enhance virological suppression rates in the country.

This review discovered that low patient adherence to ART had a notable negative impact on virological suppression in Ethiopia. This finding aligns with another meta-analysis that demonstrated a correlation between higher adherence rates and increased levels of viral suppression [19, 50], which suggests that identifying and addressing barriers to adherence are crucial to enhancing virological suppression.

Nutritional status, as indicated by BMI and baseline CD4 count, was identified as another contributing factor to viral suppression in Ethiopia. Patients with a normal BMI (18.5–24.9 kg/m^2^), according to the WHO classification, were more likely to achieve viral load suppression, which is in line with the findings of primary studies [19]; this is because BMI has a predictive impact on immune recovery, which is crucial for controlling viral replication. Additionally, the baseline CD4 count of patients was found to determine virological suppression, which is consistent with previous studies [20, 21]. CD4 count plays a vital role in immune function, protecting the body against opportunistic infections and multiplication of the virus.

Disclosure of HIV status was an important factor contributing to viral suppression in this review, and this finding is supported by other studies [32, 34, 38]; this is because disclosure improves adherence to ART [51]. This implies that encouraging environments supportive of social disclosure can enhance the viral suppression rate.

The presence of opportunistic infections plays a significant role in determining the virological suppression of patients receiving ART. Patients without any opportunistic infections are more likely to achieve virological suppression, which is supported in the literature [20, 36]. Initiating ART can help prevent many opportunistic infections, but in Ethiopia, the late presentation of patients to care and treatment leads to a high incidence of opportunistic infections and nonsuppression of the virus. This finding suggests that preventing and managing common opportunistic infections can improve viral suppression in Ethiopia. Additionally, a high baseline viral load copy reduces the rate of viral suppression in Ethiopia, which is consistent with the literature [26, 27, 36] because a high viral load indicates disease progression and adherence issues, resulting in lower viral suppression. Furthermore, limited appropriate action is taken based on viral load results in various resource-poor settings [52].

## CONCLUSIONS

In this study, the pooled percentage of virological suppression was very low compared with the global target of viral suppression by 2025 and the recent EPHI report in Ethiopia. Adherence to ART, body mass index, baseline CD4 count, disclosure, opportunistic infections, and baseline viral load count were identified as significant contributing factors to virological suppression. The current study recommends that responsible stakeholders in HIV programs should maximize their effort to achieve the global target of virological suppression by addressing significant predictors. Appropriate action on viral load test results should be taken to track progress toward ending HIV public health threats. Although sufficient primary studies were utilized to determine the pooled virological suppression rate, there was a lack of representation from certain regions of Ethiopia. As a result, it is necessary for researchers to conduct additional studies in these regions that were not included in the systematic review and meta-analysis of the current study, and thorough investigation should be conducted to determine the possible cause of temporal variation in the viral suppression rate.

## Supplementary Material

ofae168_Supplementary_Data

## Supplementary Data

Supplementary materials are available at *Open Forum Infectious Diseases* online. Consisting of data provided by the authors to benefit the reader, the posted materials are not copyedited and are the sole responsibility of the authors, so questions or comments should be addressed to the corresponding author.
