# Supplementary material for: Virological Suppression and its Predictors Among HIV/AIDS Patients on Antiretroviral Therapy in Ethiopia: Systematic Review and Meta-analysis
Source: Open Forum Infect Dis. 2024 Mar 21;11(4):ofae168. doi: 10.1093/ofid/ofae168 (PMC11036161; doi:10.1093/ofid/ofae168)
Supplement: ofae168_Supplementary_Data [file ofae168_supplementary_data.zip › PRISMA research checklist.docx]

| Table S 1:- PRISMA checklist for systematic review and meta-analysis of virological suppression and associated factors in Ethiopia | | | |
| --- | --- | --- | --- |
| **Section and Topic** | **Item #** | **Checklist item** | **Location where item**  **is reported** |
| **TITLE** | | |  |
| Title | 1 | Virological suppression rate and its predictors among HIV/AIDS patients on antiretroviral therapy before and during the COVID-19 pandemic in Ethiopia: Systematic review and meta-analysis | 1 |
| **ABSTRACT** | | |  |
| Abstract | 2 | this review aims to determine the pooled virological suppression rate and identify the pooled effect of contributing factors of viral suppression for HIV-positive patients on antiretroviral therapy in Ethiopia  Method: We searched websites and databases, including online repositories, to obtain primary studies. Publication bias was checked using Egger’s regression test, the heterogeneity of the studies was assessed using I-squared statistics and Q statistics. The Der Simonian Laird random-effects model was used to estimate the overall proportion of viral suppression  Result: A total of 21 eligible articles were used for this quantitative synthesis. The overall pooled virological suppression was 71% (95% CI: 64%, 77%). The pooled of effect of having disclosure, normal range BMI, CD4 count>=200cells/mm3 and absence of opportunistic infection had showed positive association with viral suppression; whereas the pooled effect of high viral load and poor adherence to ART had showed negative association with viral suppression | 2 |
| **INTRODUCTION** | | |  |
| Rationale | 3 | The variations in the reports of the individual study highlight the need for nationally representative data on virological suppression in Ethiopia. It is crucial to continue concerted efforts to address the uneven distribution of HIV infection and work toward achieving global targets uniformly within the remaining timeframe | 2and 3 |
| Objectives | 4 | 1. Determine the pooled virological suppression rate in Ethiopia 2. Identify the pooled effect of contributing factors of virological suppression in Ethiopia | 3 |
| **METHODS** | | |  |
| Eligibility criteria | 5 | Those studies reported the proportion of viral suppression at various time intervals and included adjusted effect measures of factors associated with virological suppression, the studies had to be conducted in Ethiopia and written in English. Studies that lacked full-text access and requiring subscription, qualitative studies, and conference proceedings without full-text reports were excluded from the analysis | 4 |
| Information sources | 6 | Web site searches (Google, Google Scholar and registries) and database searches (PubMed, Science Direct and Hinari (research4life)) were searched to find research articles. In addition, the Ethiopian Universities online repository library (University of Gondar, Addis Ababa University, Hawassa University and Bahirdar University) was searched | 4 |
| Search strategy | 7 | The search strategy used in PubMed was [((virological) OR (viral)) AND ((suppression) OR (re-suppression)) AND ((predictors) OR (factors) OR (determinants)) AND ((HIV) OR (AIDS)) AND (("antiretroviral therapy") OR (ART) OR (HAART)) AND (Ethiopia)]; similar search terms were used for Hinari | 4 |
| Selection process | 8 | Titles, abstract and full text were use to screen the studies based on the inclusion criteria. Three authors were independently involved to screen the studies. | 5 |
| Data collection process | 9 | Data extraction template was prepared by MS excel, two authors (AM and HY) were independently extract the data. The extracted data included the first author and year of publication, sample size, number of individuals with the outcome of interest, study design, study population with sample size, geographical location, funding information, and response rate. | 5 |
| Data items | 10a | The primary outcome was virological suppression. All studies reporting virological suppression was included in this review | 5 |
| Study risk of bias assessment | 11 | Two authors (AM, YT) independently evaluated the quality of the articles using the Newcastle‒Ottawa Scale, which is a tool for assessing the quality of cross-sectional, case‒control, and cohort studies (23). The criteria for cross-sectional studies consisted of three sections. The first section focused on selection and was rated on a scale of up to five stars. The second section assessed the comparability of the study and was rated on a scale of up to two stars. The third section emphasized the outcome and was rated on a scale of up to three stars. For case-control studies, the criteria included selection, which was evaluated with a maximum of four stars, comparability, assessed with a maximum of two stars, and exposure, graded with a maximum of four stars. Cohort studies had criteria that included selection, graded with up to six stars, comparability, graded with up to two stars, and outcome, graded with up to five stars. | 5 |
| Effect measures | 12 | Proportion or percentage was used to estimate the primary outcome variable | 6 |
| Synthesis methods | 13a | Crossectional and cohort studies were eligible for meta-analysis of virological suppression, whereas Crossectional studies, case control and cohort studies were used to pooled the effect of contributing factors of virological suppression | 6 |
|  | 13b | The Der Simonian Laird random-effects model was used to estimate the overall proportion of viral suppression. | 6 |
|  | 13c | Figures like forest plot and tables were used to present the finding of this systematic review and meta-analysis | 6 |
|  | 13d | Meta analysis was conducted to determine the pooled effect measures, The heterogeneity of the studies was assessed using I^2^ and Q statistics, and sensitivity analysis was performed to identify any outlier results in the included studies | 6 |
|  | 13e | Publication bias was assessed using the funnel plot and Egger's test for a more objective evaluation | 6 |
|  | 13f | Describe any sensitivity analyses conducted to assess robustness of the synthesized results. | 6 |

| **Section and Topic** | **Item #** | **Checklist item** | **Location where item**  **is reported** |
| --- | --- | --- | --- |
| **RESULTS** | | |  |
| Study selection | 16a | PRISMA flow diagram was used for overall selection process | 7 |
|  | 16b | Some students were cited but excluded for meta-analysis because its different method of outcome measurement | 7 |
| Study characteristics | 17 | Cite each included study and present its characteristics. |  |
| Risk of bias in studies | 18 | Present assessments of risk of bias for each included study. |  |
| Results of individual studies | 19 | Presented using forest plot |  |
| Results of syntheses | 20a | For each synthesis, briefly summaries the characteristics and risk of bias among contributing studies. | 8 |
|  | 20b | Present results of all statistical syntheses conducted. If meta-analysis was done, present for each the summary estimate and its precision (e.g. confidence/credible interval) and measures of statistical heterogeneity. If comparing groups, describe the direction of the effect. | 9 |
|  | 20c | Present results of all investigations of possible causes of heterogeneity among study results. | 10 |
| Reporting biases | 21 | Present assessments of risk of bias due to missing results (arising from reporting biases) for each synthesis assessed. | 11 |
| Certainty of evidence | 22 | Present assessments of certainty (or confidence) in the body of evidence for each outcome assessed. | 12 |
| **DISCUSSION** | | |  |
| Discussion | 23a | Provide a general interpretation of the results in the context of other evidence. | 21,22 |
|  | 23b | Discuss any limitations of the evidence included in the review. | 21,22 |
|  | 23c | Discuss any limitations of the review processes used. | 21,22 |
|  | 23d | Discuss implications of the results for practice, policy, and future research. | 23 |
| **OTHER INFORMATION** | | |  |
| Registration and protocol | 24a | **Prospero registration number and google link**: CRD42023434248. https://www.crd.york.ac.uk/prospero/#recordDetails  Registered by: Dagnachew Melak | 2 |
| Support | 25 | Describe sources of financial or non-financial support for the review, and the role of the funders or sponsors in the review. |  |
| Competing interests | 26 | All author declares that no conflict of interest |  |
| Availability of data, code and other materials | 27 | template data collection forms; data extracted from included studies; data used for all analyses; analytic code; any other materials used in the review are not publicly available but can be found at corresponding author upon justifiable request. |  |

*From:* Page MJ, McKenzie JE, Bossuyt PM, Boutron I, Hoffmann TC, Mulrow CD, et al. The PRISMA 2020 statement: an updated guideline for reporting systematic reviews. BMJ 2021;372:n71. doi: 10.1136/bmj.n71

For more information, visit: <http://www.prisma-statement.org/>
