# Supplementary material for: Virological Suppression and its Predictors Among HIV/AIDS Patients on Antiretroviral Therapy in Ethiopia: Systematic Review and Meta-analysis
Source: Open Forum Infect Dis. 2024 Mar 21;11(4):ofae168. doi: 10.1093/ofid/ofae168 (PMC11036161; doi:10.1093/ofid/ofae168)
Supplement: ofae168_Supplementary_Data [file ofae168_supplementary_data.zip › Quality appraisal for the included studies.docx]

| Table S 2:- Quality appraisal for cohort, case control and Crossectional studies using Newcastle‒Ottawa Scale for the included studies to systematic review and meta-analysis | | | | | | | | | | |
| --- | --- | --- | --- | --- | --- | --- | --- | --- | --- | --- |
| **Quality appraisal for cohort study using Newcastle‒Ottawa Scale** | | | | | | | | | |  |
|  | **selection (measured by six star)** | | | | **Comparability (2 star)** | **outcome (measured by five star)** | | |  |  |
| **Author** | **Representativeness of the exposed cohort** | **Selection of the non-exposed cohort** | **Ascertainment of exposure** | **Demonstration that outcome of interest was not present at start of study** | **Comparability of cohorts on the basis of the design or analysis** | **Assessment of outcome** | **Was follow-up long enough for outcomes to occur** | **Adequacy of follow up of cohorts** | **total agreed score/ 13** | **Agreed Quality Rank** |
| Ali JH et al | ** | * | * | * | ** | ** | * | * | 11 | High |
| Atnafu GT et al | ** | * | * | * | ** | * | * | * | 10 | High |
| Diress G et al | ** | * | * | * | * | * | * | * | 9 | Medium |
| Erjino E et al | ** | * | * | * | ** | * | * | * | 10 | High |
| Minyichil B | ** | * | * | * | * | * | * | * | 9 | Medium |
| Wedajo S et al | ** | * | * | * | ** | ** | * | * | 12 | High |
| Melak D et al | ** | * | * | * | * | ** | * | * | 10 | High |
| Hussen S et a | * | * | * | * | * | * | * | * | 8 | Medium |
| Sosna M | ** | * | * | * | * | * | * | * | 9 | Medium |
| **Quality appraisal for case control study using Newcastle‒Ottawa Scale** | | | | | | | | | |  |
|  | **selection (measured by four star)** | | | | **Comparability (2 star)** |  |  |  |  |  |
| **Author** | **Is the case definition adequate** | **Representativeness of the cases** | **Selection of Controls** | **Definition of Controls** | **Comparability of cases and controls on the basis of the design or analysis** | **Ascertainment of exposure** | **Same method of ascertainment for cases and controls** | **Non-Response rate** | **total score/10** | **Quality Rank** |
| Jaleta.F et al | * | * |  | * | ** | * | * | * | 8 | Medium |
| Dires YM et al | * | * |  | * | ** | ** | * | * | 9 | High |
| **Quality appraisal for Crossectional study using Newcastle‒Ottawa Scale** | | | | | | | |  |  |  |
|  | **Selection (Maximum 5 stars** | | | | **Comparability (Maximum 2 stars)** | **Outcome (Maximum 3 stars)** | |  |  |  |
| **Author** | **Representativeness of the exposed cohort** | **Sample size** | **Non-respondents** | **Ascertainment of the exposure (risk factor)** | **The subjects in different outcome groups are comparable, based on the study design or analysis. Confounding factors are controlled** | **Assessment of outcome** | **Statistical test** |  | **Agreed total score/10** | **Quality rank** |
| Anito AA et a | * | * | * | * | * | ** | * |  | 8 | High |
| Berihun H et a | * | * | * | * | ** | ** | * |  | 9 | High |
| Fenta DA | * |  | * | * | * | ** | * |  | 7 | Medium |
| Kolako E | * | * | * | * | * | ** | * |  | 8 | High |
| Melku M et al | * | * | * | * | ** | ** | * |  | 9 | High |
| Sado AG et a | * | * | * | * | * | ** | * |  | 9 | High |
| Sorsa A | * |  | * | * | * | ** | * |  | 7 | Medium |
| Waju B et al | * | * | * | * | ** | ** | * |  | 9 | High |
| Desta A et al | * |  | * | * | ** | ** | * |  | 8 | High |
| Haile B et al | * | * | * | * | ** | * | * |  | 8 | High |
